# Parental Perceptions of the Social Environment Are Inversely Related to Constraint of Adolescents’ Neighborhood Physical Activity

**DOI:** 10.3390/ijerph13121266

**Published:** 2016-12-21

**Authors:** Maura Kepper, Stephanie Broyles, Richard Scribner, Tung-Sung Tseng, Jovanny Zabaleta, Lauren Griffiths, Melinda Sothern

**Affiliations:** 1 Contextual Risk Factors Laboratory, Pennington Biomedical Research Center, 6400 Perkins Avenue, Baton Rouge, LA 70808, USA; stephanie.broyles@pbrc.edu; 2 Behavioral & Community Health Sciences Department, School of Public Health, Louisiana State University Health Sciences Center, New Orleans, LA 70112, USA; ttseng@lsuhsc.edu (T.-S.T.); jzabal@lsuhsc.edu (J.Z.); lgri10@lsuhsc.edu (L.G.); msothe@lsuhsc.edu (M.S.); 3 Department of Epidemiology, School of Public Health, Louisiana State University Health Sciences Center, New Orleans, LA 70112, USA; rscrib@lsuhsc.edu; 4 Stanley S. Scott Cancer Center, Louisiana State University Health Sciences Center, New Orleans, LA 70112, USA; 5 Department of Pediatrics, School of Medicine, Louisiana State University Health Sciences Center, New Orleans, LA 70112, USA

**Keywords:** social environment, adolescent outdoor play, parenting behaviors, physical activity

## Abstract

Background: The current study examined relationships between the neighborhood social environment (parental perceived collective efficacy (PCE)), constrained behaviors (e.g., avoidance or defensive behaviors) and adolescent offspring neighborhood physical activity in low- versus high-incivility neighborhoods. Methods: Adolescents (*n* = 71; 11–18 years (14.2, SD ± 1.6); male = 37 (52%); non-white = 24 (33.8%); low-income = 20 (29%); overweight/obese = 40 (56%)) and their parents/guardians enrolled in the Molecular and Social Determinants of Obesity in Developing Youth study were included in the current study. Questionnaires measured parents’/guardians’ PCE, constrained outdoor play practices and offspring neighborhood physical activity. Systematic social observation performed at the parcel-level using Google Street View assessed neighborhood incivilities. *t*-tests and chi-square tests determined differences by incivilities. Multilevel regression models examined relationships between PCE and: (1) constrained behaviors; and (2) neighborhood physical activity. The Hayes (2013) macro determined the mediating role of constrained behaviors. Results: Parents who had higher PCE reported lower levels of avoidance (*p* = 0.04) and defensive (*p* = 0.05) behaviors. However, demographic variables (i.e., gender, race and annual household income) limited these results. The direct relationship between PCE and parent-reported neighborhood physical activity was statistically significant in high-incivility neighborhoods only. Neither avoidance nor defensive behavior mediated the relationship between PCE and neighborhood physical activity. Conclusions: PCE influences parenting behaviors related to youth physical activity. Community-based programs that seek to facilitate social cohesion and control may be needed to increase adolescents’ physical activity.

## 1. Introduction

Physical inactivity is a major contributing factor to the obesity epidemic, and adolescence is associated with a decline in physical activity [1]. Despite known health benefits, a large portion of the United States (U.S.) adolescent population does not engage in the recommended 60 min of accumulated moderate to vigorous physical activity (MVPA) daily [2,3,4]. Of particular concern is that physical activity levels decline during the transition from childhood to adolescence, a period crucial to the development and creation of habitual participation in physical activity throughout the life course [3]. One prominent explanation of inactivity is that adolescents are spending too little time playing outdoors due to social and physical neighborhood factors [5,6]. It is possible that if parents restrict outdoor play due to concerns about their neighborhood, their adolescent offspring will be less active overall, as time spent outdoors is positively associated with overall physical activity [1]. Generally, individuals in more disadvantaged neighborhoods have lower levels of physical activity and higher rates of obesity, even when controlling for individual-level socioeconomic status (SES). These relationships may be due to safety concerns (crime, victimization, poorly lighted streets); the physical environment (incivilities, access to parks/playgrounds, sidewalks, walkable destinations); or to differences in neighborhood social environment (perceived safety, social cohesion, social capital, social support, perceived collective efficacy) [5,6,7,8,9,10]. 

Emerging social environmental research identifies perceived collective efficacy as a potentially influential determinant of physical activity opportunities for both adults [11,12,13] and youth [6,10]. Collective efficacy is a form of social capital and is defined as a measure of perceived social cohesion (mutual trust among neighborhoods) and social control (capacity and willingness of the group to intervene for a common goal) [14,15,16]. Numerous social capital indicators, including collective efficacy, have been identified and tested in relation to health behaviors and outcomes, yet have resulted in mixed conclusions [7]. For example, physical activity studies operationalizing the social environment as sense of belonging, social cohesion or norms of reciprocity illustrate no significance, whereas studies assessing collective efficacy report significant results [9,17]. Furthermore, the majority of studies relating the social environment and physical activity assessed the impact on active transport, rather than outdoor play in general and were performed mainly in older populations [10]. 

While a number of studies have assessed the impact of the social environment on children’s physical activity, few studies have considered physical activity-related parenting behaviors (e.g., constrained behaviors), which may ultimately restrict or facilitate adolescents’ physical activity [1]. Adolescents who are free to play outdoors and travel actively without adult supervision accumulate more physical activity than those who are not; therefore understanding whether parental perceptions of their neighborhood impact physical activity-related parenting behaviors may be crucial to improving overall activity among adolescents [18]. Constrained behavior is defined as the act of restricting offspring’s physical activity and is categorized as “avoidance” or “defensive” behaviors depending on whether physical activity was avoided or modified, respectively. Examples of avoidance behavior include parents driving their children to school instead of allowing them to walk or forbidding unsupervised outdoor play. Examples of defensive behavior include parental accompaniment while walking to school or restriction of outdoor play to the backyard [1]. Carver and colleagues (2010) is the only study to examine environmental influence on constrained behaviors and found that perceived risk of harm to a child in the neighborhood was positively associated with constrained behavior resulting in lower levels of active transport and MVPA outside of school hours for children and adolescents [1]. However, no studies to our knowledge have explored the influence of parental perceived collective efficacy on constrained behaviors, nor have any further explored the impact of the physical environment on parental perceptions and behaviors impacting adolescents’ activity.

**Figure 1:**
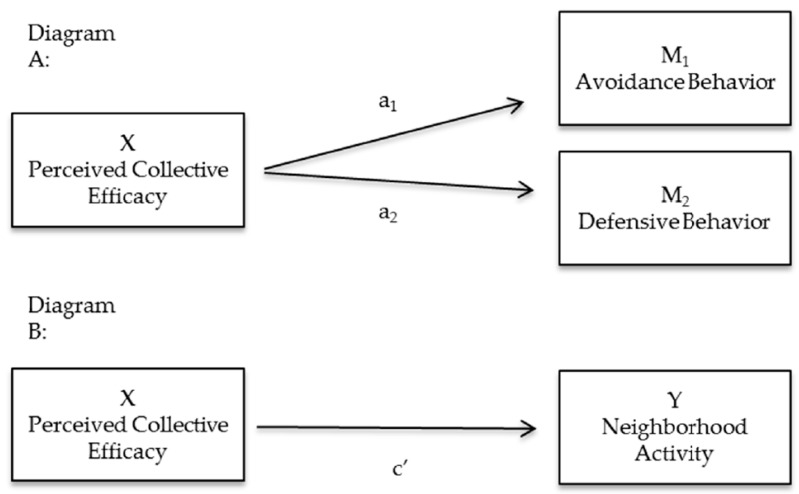
Path diagrams for regression analyses between perceived collective efficacy and (1) constrained behavior (a_1_ avoidance behavior, a_2_ defensive behavior) and (2) c’ neighborhood activity.

**Figure 2:**
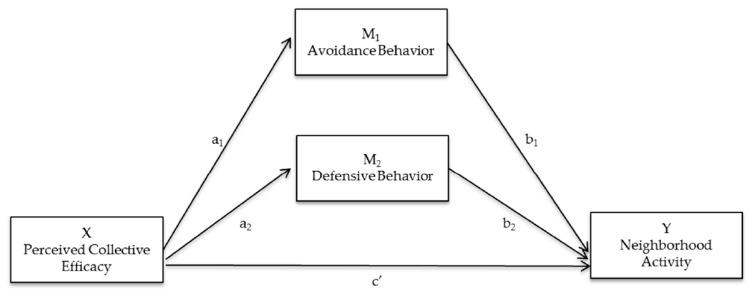
Path diagram for a parallel multiple mediator model with two mediators (a quantifies how much two cases that differ by one unit on X are estimated to differ on M; b quantifies how much two cases that differ by one unit of M but that are equal on X differ by b units on Y; c’ indicates the direct effect of X on Y. c’ quantifies how much two cases that differ by one unit on X are estimated to differ on Y [22].

The physical environment (e.g., neighborhood incivilities) may be the foundation for, or etiology of, perceived collective efficacy and may ultimately explain differences in perceptions of collective efficacy among parents living in low- versus high-incivility neighborhoods. The social disorganization theory [19] and the norms and collective efficacy model [20] posit that characteristics of the built environment influence the ability of community members to establish cohesive relationships and create a shared set of socially-accepted norms that promote the willingness to intervene on behalf of the common good [21]. Therefore, achieving the recommended 60 min of daily physical activity is difficult due to a complex interaction among the physical environment, parental perceptions of the social environment and their constraints on adolescent outdoor play. Additional studies are necessary to understand the influence of the social environment on parenting behaviors and offspring’s physical activity, especially among populations who live in high-incivility neighborhoods. The current study examined relationships between parental perceived collective efficacy, constrained behaviors (e.g., avoidance or defensive behaviors) and adolescent offspring neighborhood physical activity in low- versus high-incivility neighborhoods. Conceptual path diagrams are presented in Figure 1 and Figure 2. 

## 2. Materials and Methods

### 2.1. Study Participants

Adolescents (*n* = 71) in the Molecular and Social Determinants of Obesity in Developing Youth (MSDO) study were accessed for the current study. Participants were recruited from an existing cohort of healthy, exclusively pre-pubertal children (*n* = 149) who previously participated in the Mechanisms for the Metabolic Syndrome in Prepubertal Youth (MET) study (2006–2010). All 149 MET participants were eligible to participate. Only those who were able to be contacted and who were willing to participate were included in the current study (*n* = 71). The characteristics of the MET cohort have been described previously [23]. Participants were included if they did not meet exclusion criteria (Tanner > 2, diagnosed with cardiovascular, metabolic or liver disease, born from a mother with gestational diabetes, immediate family with history of type 1 or 2 diabetes) during their initial enrollment into the prior MET study. 

### 2.2. Procedures

Parents and adolescents who expressed interest in the study and met eligibility criteria were invited to participate in one study visit at the Louisiana State University Health Sciences Center (LSUHSC) Clinical and Translational Research Center (CTRC) at University Medical Center in New Orleans. A parent/guardian provided informed consent, and each participant gave written assent prior to enrollment. The study was conducted in accordance with the Declaration of Helsinki and was approved by the LSUHSC and CTRC Institutional Review Boards (Ethical Approval #8143). During the visit, adolescents participated in a physical exam by trained pediatric nurses. The adolescent and his/her parent/guardian completed self-administered questionnaires, and an accelerometer was sent home to be returned via a pre-paid envelope approximately one week after the visit. 

### 2.3. Measurements

#### 2.3.1. Perceived Collective Efficacy

Perceived collective efficacy was assessed using a survey instrument modified from the 1995 Community Survey of the Project on Human Development in Chicago Neighborhoods [10]. Items assessed two constructs: (1) perceptions of cohesion, the level of trust and attachment among neighbors; and (2) social control, a belief in the capacity of neighborhood residents to intervene to reach a collective goal [11]. Parents/guardians were asked to indicate their level of agreement with 5 statements assessing how cohesive they felt their neighborhoods were, such as “people around here are willing to help their neighbors” and “this is a close-knit neighborhood”. The parent/guardian chose from 5 response options with subsequent coding (in parenthesis): strongly disagree (1); disagree (2); neither agree nor disagree (3); agree (4); or strongly agree (5). Cohesion was summarized as the mean across all 5 items that assessed the parent/guardian’s perception of neighborhood cohesion (Cronbach’s alpha = 0.69). Six statements assessed the perception of social control based on how likely the parent/guardian thought that neighbors would intervene in certain situations (e.g., “if children were skipping school and hanging out on the street corner” or “if children were spraying painting graffiti on a local building”). The parent/guardian chose from 5 response options with subsequent coding (in parenthesis): very unlikely (1); unlikely (2); neither likely nor unlikely (3); likely (4); or very likely (5). Social control was summarized as the mean across all 6 items that assessed the parent/guardian’s perception of neighborhood control (Cronbach’s alpha = 0.89). Collective efficacy was calculated as the mean of cohesion and control summary variables. A higher score indicates higher levels of perceived collective efficacy (mean (SD): 4.0 (0.6); range: 2.3–5.0). This method was applied from Sampson and colleagues (1997) [24]. 

#### 2.3.2. Constrained Outdoor Play Practices

Constrained outdoor play practices were assessed using a questionnaire adapted from Ferraro’s (1995) [25] indices of constrained behavior with regard to crime victimization and was further validated by Carver and colleagues (2010) [1]. To assess avoidance behavior, parents/guardians were asked to indicate their level of agreement with 9 statements about protecting their offspring’s engagement in various outdoor activities in their neighborhood. Similarly, to assess defensive behavior, parents/guardians were asked to indicate their level of agreement with 6 statements about defensive measures taken regarding their offspring’s engagement in various outdoor activities in their local neighborhood. Response options and subsequent coding (in parenthesis) were: strongly disagree (−2); disagree (−1); neither/don’t know (0); agree (1); strongly agree (2). All responses were summed to compute overall avoidance (possible range: −18–18; Cronbach’s alpha = 0.80) and defensive behavior (possible range: −12–12; Cronbach’s alpha = 0.72) scores, with a higher number indicating more avoidance or defensive behavior, respectively. Carver et al. (2010) found this survey to have moderate to high internal consistency for each score and moderate test-retest reliability when used in children aged 15–17 years [1]. 

#### 2.3.3. Neighborhood Physical Activity

A self-administered questionnaire asked parents/guardians how often (never, once a month or less, once every other week, once a week, two or three times per week, or four times per week or more) their child is physically active in a series of places in their neighborhood (i.e., in their yard, in their street, in a near-by park or playground, etc.). These ordinal responses were rescaled to indicate the number of times per month each child was active in specific neighborhood locations. This number was then dichotomized to active versus not active in the neighborhood, based on the median value (being active in the neighborhood 25 times per month). The median value was approximated to the nearest whole number. Methods were adapted from Tappe and colleagues (2013) who reported high test-retest reliability and internal consistency [26]. 

Adolescent physical activity was also assessed objectively using GT3M ActiGraph triaxial accelerometers (Manufacturing Technologies Inc., Health Systems, ActiGraph, Fort Walton Beach, FL, USA). Study participants wore the accelerometer around their waist for a minimum of 3 days (72 h) and a maximum of 7 (168 h) and were allowed to remove the accelerometer during sleep [27]. Data were treated using a 24-h protocol to determine nocturnal sleep period and a separate non-wear algorithm previously published by Tudor-Locke and colleagues (2014) [28]. Valid cases were defined as having ≥3 days with ≥8 h of waking wear time in a 24-h period. Only valid cases (*n* = 37) were used for the analysis of Evenson (2008) cut points into sedentary, light, moderate and vigorous physical activity [29]. 

#### 2.3.4. Weight Status

An electronically-calibrated scale (Indiana Scale, Terahuat, IN, USA) and a calibrated stadiometer (Holtain, Ltd., Dyfed, UK) were used to obtain the weight (kg) and height (cm) of each study participant, respectively. BMI was calculated using the following formula: weight in kilograms/height in m^2^. BMI z-scores were calculated using the Centers for Disease Control and Prevention’s (CDC) program for calculating BMI percentiles and z-scores based on a child’s sex and age for BMI, weight and height, based on the CDC 2000 growth charts. Weight status was categorized as obese (BMI percentile ≥ 95), overweight (BMI percentile <95 and ≥85) and healthy weight (BMI percentile < 85) using BMI z-scores. 

#### 2.3.5. Incivilities

Systematic social observation (SSO) performed at the parcel-level using virtual applications (Google Street View) was used to collect data on four major components of neighborhood incivilities: physical disorder, physical decay, perceived safety and street safety. This method was previously proven reliable compared to street-level SSO and direct observations performed by raters traveling to the neighborhood [30]. Observations were performed over 6 months in August 2015–January 2016. Google Street View images were dated from 2011–2015. 

Physical disorder: A principal component analysis was used to determine whether the following items denoted physical disorder: (1) garbage/litter on the street, in residential yards, commercial, businesses, institutional, industrial lots (rated 0–4: none, light, moderate, heavy); (2) graffiti or graffiti that had been painted over on buildings or signs (rated no (0), yes (1)); and (3) residential porches that were cluttered with personal items (rated no (0), yes (1)). However, no clear factor emerged (Cronbach’s alpha = 0.3), and items were instead analyzed individually (Table 1). Ultimately, garbage/litter was considered as a marker for physical disorder due to its having greater variation (mean (SD): 2.3 (3.2); min, max: 0.0, 19.0), whereas there were few streets with graffiti/graffiti that had been painted over (6.6% of streets) or cluttered porches (16.3% of streets). 

Physical decay: A principal component analysis was used to identify whether the following items denoted physical decay: (1) condition (rated 1–3: well-kept/good, fair, poor/badly deteriorated) of residential units; (2) condition (rated 1–3: well-kept/good, fair, poor/badly deteriorated) of residential yards; and (3) the presence of abandoned cars (rated no (0), yes (1)). A physical decay scale (Cronbach’s alpha = 0.6) was created by summing all of the items, which resulted in a greater value indicating worse physical decay (mean (SD): 3.1 (1.6); min, max: 1, 12; Table 1).

Safety: A principal component analysis was used to identify whether the following items denoted safety: (1) raters’ perceptions of whether the neighborhood was a “safe place to live”; (2) raters’ perceptions of whether they would feel “safe walking at night” (rated 1–5; definitely safe to definitely unsafe); and (3) whether the rater felt they could live in the neighborhood (rated 1–5; strongly agree to strongly disagree). A safety scale (Cronbach’s alpha = 0.8) was created by summing all items with a greater value, which indicated a less safe environment (mean (SD): 4.9 (2.3); min, max: 2, 12; Table 1). 

Street safety: A principal component analysis was used to identify whether the following items accurately denoted street safety: (1) the presence of speed limit signs (rated no (0), yes (1)); (2) presence of a bike lane (rated no (0), yes (1)); (3) lighting (rated 1–4: >75%, 50%–74%, 25%–49%, <25%); and (4) traffic volume (rated 1–4; no traffic, light, moderate, heavy). However, no clear factor emerged (Cronbach’s alpha = −0.3), and items were instead analyzed individually (Table 1). Ultimately, traffic and lighting were considered in markers of street safety. The presence of a bike lane was not included as an item indicating street safety because no streets contained bike lanes.

Low- versus high-incivility neighborhoods: A principal component analysis was used to create an overall incivilities summary variable from the following variables: (1) garbage/litter on the street; (2) physical decay summary scale; (3) safety summary scale; (4) traffic volume; and (5) lighting on the street. The factor included: (1) garbage/litter on the street; (2) physical decay summary scale; and (3) safety summary scale with an eigenvalue of 2.2 (Table 1). The factor was then dichotomized at the median value (−0.28) to create low- and high-incivility groups.

### 2.4. Data Analysis

Statistical analyses were performed using SAS Version 9.4 (SAS Institute Inc., Cary, NC, USA). A *p*-value < 0.05 was considered statistically significant. Descriptive statistics (means, standard deviations, frequencies as appropriate) were calculated for demographic and anthropometric characteristics. Unpaired *t*-tests and chi-square tests were used to test differences between low- and high-incivility neighborhoods. 

Multilevel linear (SAS function: PROC MIXED) or logistic (SAS function: PROC GLIMMIX) models, which included households as a random effect to account for adolescents living in the same household, were used to examine the relationship of perceived collective efficacy and the outcomes of interest (parental constrained outdoor play practices and adolescent’s physical activity) (Figure 1). Analyses included both parent-reported neighborhood physical activity and objectively-measured MVPA as physical activity outcomes; however, due to the small sample size of objective measures (*n* = 37), only parent-reported neighborhood physical activity is presented. First, univariate models were run for all potential correlates. Next, multivariable models were analyzed using multilevel general linear (PROC MIXED) and logistic (PROC GLIMMIX) regression models, controlling for demographic variables (gender, race and annual household income). Furthermore, multilevel regression analyses, controlled for demographic variables and BMI z-score, were analyzed by incivilities to examine the relationship between perceived collective efficacy and all proposed outcomes (parental constrained outdoor play practices and adolescent’s physical activity) within low- and high-incivility neighborhoods. The Kenward-Roger approximation (SAS function: DDFM = KR) was used to calculate the degrees of freedom [30]. 

The publically available Hayes (2013) PROCESS macro [31] was implemented to determine whether constrained outdoor play practices mediated the relationship between parental perceived collective efficacy and adolescent physical activity (Figure 2). Mediation models were run overall and within low- and high-incivility neighborhoods. A parallel multiple mediator model with 95% confidence intervals derived from 1000 bootstrap resamples was used in order to include both avoidance and defensive behavior as potential mediators. Mediation models controlled for gender, race and annual household income.

## 3. Results

### 3.1. Descriptive Statistics

The study participants ranged in age from 11–18 years (mean (SD): 14.2 (1.6)). Approximately half (52.1%) of the participants were male (Table 2). The majority of the participants were white (77.2%) and high-income (71.4%) (Table 2). More than half of the participants were either obese or overweight (56.4%), as follows: (1) 40.9% of participants were obese; (2) 15.5% of participants were overweight; and (3) 43.6% of participants were healthy weight. 

On average, parents/guardians reported that their child was active in locations around their neighborhood 18 times per month (Table 2). Valid accelerometry data were available in 47.9% (*n* = 37) of the sample. Accelerometry data indicated only 25.7 (13.7) (mean (SD)) minutes of overall moderate-to-vigorous physical activity (MVPA) per day and 21.0 (14.6) (mean (SD)) minutes of MVPA outside of school hours per day (weekdays only). Overall, parents reported high levels of perceived collective efficacy (mean (SD): 4.0 (0.6)) with a possible range of one (low perceived collective efficacy) to 5.0 (high perceived collective efficacy).

### 3.2. Differences in Low- versus High-Incivility Neighborhoods

Neither parent-reported nor objectively measured physical activity differed between adolescents living in low- versus high-incivility neighborhoods. However, participants who lived in high-incivility neighborhoods (42.3%) had a significantly higher BMIz score (mean (SD): 1.4 (0.8)) than participants from low incivility-neighborhoods (mean (SD): 0.9 (1.1); *p* = 0.03; Table 2). Furthermore, parents who lived in high-incivility neighborhoods reported more avoidance (mean (SD): −3.3 (7.7)) and defensive behavior (mean (SD): −1.3 (5.5)) compared to parents who lived in low-incivility neighborhoods (mean (SD): −7.4 (6.0), −3.8 (3.6); *p* = 0.01, *p* = 0.02, respectively; Table 2). Parents who lived in high-incivility neighborhoods also reported lower levels of perceived collective efficacy (mean (SD): 3.8 (0.7)) compared to parents who lived in low-incivility neighborhoods (mean (SD): 4.1 (0.4); *p* = 0.03; Table 2).

### 3.3. Parental Perceived Collective Efficacy and Constrained Outdoor Play Practices (Figure 1, Diagram A)

Perceived collective efficacy was inversely associated with avoidance behavior in the univariate model (β = −3.24; *p* = 0.04; Table 3). However, in the final multivariable model controlling for demographic variables (i.e., gender, race and annual household income), this relationship did not reach the level of significance (β = −2.75; *p* = 0.07; Table 3). Similarly, perceived collective efficacy was inversely related to defensive behavior (β = −2.01; *p* = 0.05; Table 3), which suggests that parents exhibit less defensive behavior when they perceive a higher level of collective efficacy. While not statistically significant after controlling for demographic variables, perceived collective efficacy did vary in the expected direction with defensive behavior (β = −1.84; *p* = 0.08; Table 3).

In multilevel regression analyses stratified by neighborhood incivilities, relationships between perceived collective efficacy and avoidance behavior were not significant within either low- (β = −0.13; 95% CI, −5.09, 4.83; *p* = 0.96) or high-incivility (β = −2.94; 95% CI, −7.34, 1.46; *p* = 0.18) neighborhoods (Table 4). Furthermore, relationships between perceived collective efficacy and defensive behavior were not significant within either low-incivility (β = 0.04; 95% CI, −3.36, 3.45; *p* = 0.98) or high-incivility (β = −2.71; 95% CI, −6.23, −0.81; *p* = 0.12) neighborhoods (Table 4). 

### 3.4. Parental Perceived Collective Efficacy and Offspring’s Neighborhood Activity (Figure 1, Diagram B)

Relationships between perceived collective efficacy and parent-reported offspring neighborhood activity were not significant (β = 0.14; *p* = 0.19; Table 3). When controlled for demographic variables, relationships between perceived collective efficacy and neighborhood activity (β = 0.17; *p* = 0.14; Table 3) remained non-significant. Relationships to objectively measured physical activity by accelerometry were also not significant. 

Perceived collective efficacy was significantly related to neighborhood activity in high-incivility neighborhoods (β = 0.29; 95% CI, 0.04, 0.54; *p* = 0.02), but not in low-incivility neighborhoods (β = −0.06; 95% CI, −0.51, 0.38; *p* = 0.77; Table 4). However, perceived collective efficacy was not related to objectively measure physical activity in either low- or high-incivility neighborhoods. 

### 3.5. Mediated Pathways (Figure 2)

**Figure 3:**
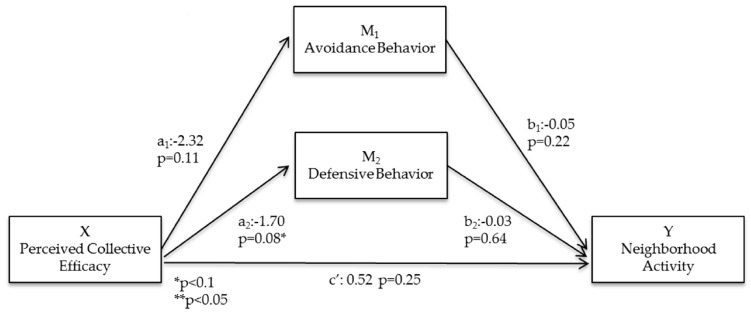
Statistical diagram of the parallel multiple mediator model for the relationship between perceived collective efficacy and neighborhood activity, controlled for demographic variables.

Neither avoidance nor defensive behavior mediated the relationship between perceived collective efficacy and neighborhood physical activity (Figure 3). Mediation results were similar when analyzed using objectively measured physical activity. As reported above, direct relationships between perceptions of collective efficacy and neighborhood physical activity were not significant (c’ = 0.52; *p* = 0.25; Figure 3). Furthermore, the direct relationships between avoidance (b_1_ = −0.05; *p* = 0.22) or defensive behavior (b_2_ = −0.03; *p* = 0.64) and neighborhood physical activity were not statistically significant (Figure 3). 

**Figure 4:**
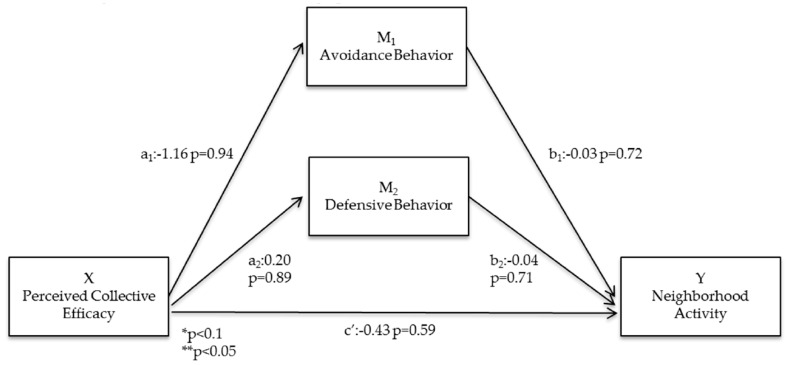
Statistical diagram of the parallel multiple mediator model for the relationship between perceived collective efficacy and neighborhood activity within low incivility neighborhoods, controlled for demographic variables.

Within low-incivility neighborhoods, neither avoidance nor defensive behavior mediated the relationship between perceived collective efficacy and neighborhood physical activity (Figure 4). The direct relationship between perceptions of collective efficacy and neighborhood physical activity were not statistically significant (c’ = −0.43; *p* = 0.59; Figure 4). The relationships between perceived collective efficacy and avoidance (a_1_ = −1.16; *p* = 0.94) and defensive behavior (a_2_ = 0.20; *p* = 0.89) were not significant with low effect sizes. Furthermore, the direct relationships between avoidance (b_1_ = −0.03; *p* = 0.72) or defensive behavior (b_2_ = −0.04; *p* = 0.71) and neighborhood physical activity were not statistically significant (Figure 4). 

**Figure 5:**
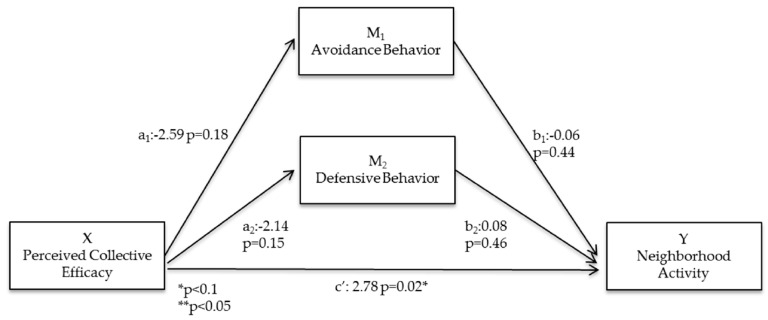
Statistical diagram of the parallel multiple mediator model for the relationship between perceived collective efficacy and neighborhood activity within high incivility neighborhoods, controlled for demographic variables.

Within high-incivility neighborhoods, neither avoidance nor defensive behavior mediated the relationship between perceived collective efficacy and neighborhood physical activity (Figure 5). However, there was a statistically-significant direct relationship between perceived collective efficacy and neighborhood physical activity in high-incivility neighborhoods (c’ = 2.78; *p* = 0.02; Figure 5). Although not statistically significant, the relationship between perceived collective efficacy and avoidance (a_1_ = −2.59; *p* = 0.18) and defensive behavior (a_2_ = −2.14; *p* = 0.15) had large effect sizes relative to the same relationship in low-incivility neighborhoods. The direct relationships between avoidance (b_1_ = −0.06; *p* = 0.44) or defensive behavior (b_2_ = 0.08; *p* = 0.46) and neighborhood physical activity were not statistically significant (Figure 5). 

## 4. Discussion

This novel study explored associations among the social and physical environment and parenting behaviors to explain anticipated low levels of physical activity in a small sample of adolescents. This preliminary study was the first to report that parents who perceived low levels of collective efficacy were more likely to constrain their adolescents’ outdoor play practices. Yet, constrained behaviors did not impact neighborhood physical activity in these offspring. Results from multilevel regression analyses by incivilities suggested that parental perceptions of collective efficacy might be more influential in high- versus low-incivility neighborhoods. Our findings are consistent with the ecological perspective and stress the importance of understanding how the social and physical environments interact to impact parenting behaviors and adolescents’ neighborhood physical activity to reduce adverse health outcomes at early ages. 

Parents who perceived higher levels of collective efficacy exhibited less avoidance and defensive behavior; however, gender, race and annual household income diminished these findings. Other studies have reported similar inverse relationships between other characteristics of the social environment (i.e., perceived risk and perceived safety) and parental constrained outdoor play practices [1,33]. Parents of children 15–17 years of age who perceived greater risk of their child being harmed in their neighborhood had higher levels of avoidance and defensive behavior [1]. In the same cohort, perceived personal safety was related to lower levels of avoidance and defensive behaviors [33]. In our sample, significant relationships between perceptions of collective efficacy and constrained outdoor play practices did not remain after controlling for demographic variables (gender, race and annual household income). This may be due to low-income families being disproportionately exposed to a host of environmental factors that may further restrict their physical activity, such as limited access to safe areas to play, that were not considered in the current study [34,35]. In our sample, a higher number of low-income and non-white participants lived in neighborhoods characterized by high incivilities compared to high-income and white participants, and collective efficacy may have been more influential in these high-incivility neighborhoods (Table 2 and Table 4). Yet, our sample was predominantly white and high income (Table 2), which was likely not an ideal sample in which to explore these relationships; moreover, the low variability in parental perceptions of collective efficacy may have limited our ability to detect significant relationships. Furthermore, parenting behaviors may be especially relevant in low-income populations, where children have less exposure to physically-active parents, fewer parental role models, less ability for parental supervision of outdoor play and less joint activity with parents [35]. Thus, a more diverse sample that better represented the racial and socio-economic composition of the urban New Orleans area may have revealed stronger correlations between parents’ perceptions of collective efficacy and constrained outdoor play practices, especially in high-incivility neighborhoods. 

Perceptions of collective efficacy were related to parenting behaviors, but direct relationships were not found between parents’ perceptions of collective efficacy and their children’s neighborhood physical activity. In contrast to our findings, numerous research studies in moderate to large child/adolescent populations reported positive associations between perceived collective efficacy and physical activity [5,6,10,34,36]. In particular, children whose mothers perceived higher levels of collective efficacy in their neighborhood played outside for longer periods of time, watched less television and visited the park or playground more frequently [10]. However, these studies were primarily performed in children under 11 years of age. Parental perceptions of collective efficacy may be less influential in older, more autonomous youth in the current study because parents/guardians (e.g., constrained outdoor play practices) may modify their actions to align with their belief that older offspring possess better self-regulatory skills. 

The present study, to our knowledge, is the first to explore how physical environmental factors (i.e., incivilities) measured objectively may alter the relationship between parents’ perceptions of collective efficacy and: (1) parental constrained outdoor play practices; and (2) adolescents’ physical activity. Individuals in our sample who lived in high-incivility neighborhoods reported lower perceived collective efficacy and more avoidance and defensive behaviors than those living in low-incivility neighborhoods (Table 2). This finding is supported by the “broken windows theory”, which states that the presence of disorder, such as graffiti, litter or abandoned homes, communicates a dangerous, unmonitored environment, which influences individual’s attitudes and perceptions [10]. Although not statistically significant, regression and mediation analyses within low- and high-incivility neighborhoods suggest that relationships between perceptions of collective efficacy and behaviors are more meaningful in neighborhoods with high incivilities. Perceived collective efficacy may have a lower impact in neighborhoods with low incivilities, since neighborhoods with appealing physical environments have additional modes of social control, such as gated entrances, security that decreases graffiti, landscape services, well-kept sidewalks and roads for play [37]. Therefore, collective efficacy may not be the key process by which parents make decisions regarding children’s outdoor play in low-incivility neighborhoods. Whereas, in neighborhoods with high incivilities, perceived collective efficacy may be a key process by which parents overcome the negative effects of incivilities, thereby increasing the importance of social cohesion and control among neighbors. This is supported by previous research indicating that the relationship between incivilities and fear of crime was mitigated by higher levels of perceived collective efficacy [37,38]. 

Several studies report relationships between the social environment and physical activity, but a paucity of research has investigated whether parenting behaviors are the mechanism by which the environment impacts children’s physical activity and health outcomes [1,5,6,10,34,36]. The current study is novel because it investigated parental constraint of outdoor play practices and how this relates to neighborhood physical activity. Results indicated that constrained outdoor play practices was not a significant mediator. This can be explained by the non-significant direct relationship between perceived collective efficacy and adolescent’s neighborhood physical activity, which ultimately precluded our ability to detect mediation. However, mediation results within high-incivility neighborhoods suggest that constrained outdoor play practices may be the mechanism by which perceived collective efficacy impacts neighborhood physical activity (Figure 5). Carver and colleagues (2010) reported that higher levels of parental constraint on outdoor play due to perceived risk of harm resulted in less active transport and lower total objectively measured MVPA outside of school hours for both children aged 10–11 years and adolescents aged 15–17 [1]. However, there were inconsistencies throughout their results. Constrained behavior influenced female, but not male adolescents’ MVPA, and hypothesized associations were true only for younger children and adolescent girls. Our results found no gender differences in constrained outdoor play practices, which may indicate that the adolescent population in the current study may be more autonomous. As stated earlier, adolescents from the current study presumably were less restricted by parenting behaviors than those reported for younger children [1]. In Carver’s study, parents of children ages 10–11 years reported more avoidance and defensive behaviors compared to those of children aged 15–17 years [1]. Adolescence is a time of increased autonomy when rules and boundaries regarding outdoor play may be re-negotiated [1,39,40]. Additionally, greater autonomy may increase the pertinence of the child’s own perception of neighborhood collective efficacy on their physical activity behaviors, especially since adolescents may experience the neighborhood context in a manner distinct from their parents [1,41,42]. Furthermore, adolescents may lose interest in specific outdoor locations in their neighborhood, such as their yard, cul de sac or local park or playground, which may result in decreased physical activity [43]. Furthermore, the lack of significant relationships between constrained behavior and physical activity may also be explained by today’s children belonging to the ‘backseat generation’ that are chauffeured to structured leisure-time activities outside of their neighborhood more than previous generations [1]. 

### Limitations

The low sample size limited several findings. Furthermore, due to the small sample and cross-sectional nature of this study, our mediation analyses are exploratory in nature. Nevertheless, our findings were able to detect a significant association between perceived collective efficacy and parental constrained behaviors. Subsequent research should examine hypothesized relationships in a larger, more diverse cohort. Regrettably, the target population, and resulting small sample size, was limited by the availability of potential volunteers (*n* = 146) who participated in the parent study, the MET study (2006–2012). The selection of this convenience group of returning study participants inadvertently generated another limitation. The MET cohort included children recruited throughout southeastern Louisiana and included primarily white families from high income households, which limits the generalizability of the findings to more diverse populations. 

The inclusion of objectively measured physical activity data is a strength of this study; however, 47.8% of participants did not achieve valid wear time (a minimum of three days with 8 h of wear time per day) and, thus, were not included for analyses. Defining valid wear time is complex, and varying definitions have resulted in a range from 38%–84% of study samples that achieved usable accelerometry data [44]. Furthermore, accelerometers were not equipped with global positioning technologies (GPS) to determine the location of physical activity. Therefore, the study relied on potentially biased parent-reported perceptions of their child’s neighborhood physical activity. Additional studies with a larger sample of valid accelerometer data that incorporates Global Positioning Systems (GPS) technology are warranted to increase the power to detect relationships between neighborhood characteristics and physical activity occurring in the neighborhood.

Although numerous factors were considered, additional factors may explain the overall low levels of physical activity in our sample. For example, participation in organized sports teams and the types of services available to youths in a neighborhood have been shown to influence adolescent’s physical activity [1]. Furthermore, individual factors (e.g., self-efficacy) or neighborhood-level factors (e.g., access to greenspace, incivilities, peers in the neighborhood and living within a walkable distance to school or businesses) that were not considered may have influenced activity levels. Additionally, future research should consider psychosocial factors (i.e., self-esteem, psychological adjustment, substance abuse) that may be influenced by parenting practices and ultimately influence physical activity levels in our sample [45,46,47]. However, a major strength of the current study was the inclusion of a novel conceptualization of the physical environment as incivilities, which were objectively measured by systematic social observation (SSO) at the parcel-level using Google Street View. Measures were aggregated to create summary measures for the street segment on which the child lived. Therefore, this unique application may not have truly captured the entire neighborhood-level incivilities. Further research is needed to determine if SSO performed on a larger area (e.g., for the entire block face or on 10% of street segments within the block group) would provide a more complete assessment of the associations among perceived collective efficacy and parenting behaviors, physical activity and obesity-related outcomes. Regardless of this limitation, the SSO method provided a more accurate overall assessment of incivilities because factors were measured at the smallest geographic unit possible (parcel-level), which potentially provides a more detailed description of neighborhoods and captures incivilities that are within an individual’s control (i.e., their residential and yard condition, litter in their yard, etc.) [30]. This may be advantageous for micro-level studies of individual health outcomes, such as those included in the current study. 

The social environment is broad; no definition or single measure encompasses its effect on an individual’s behaviors and health outcomes [7]. The way in which the social environment is measured and conceptualized produces inconsistent results when examining physical activity and health [7,48]. The current study was limited to the assessment of perceived collective efficacy. Thus, other social environmental factors (e.g., sense of belonging, trust and norms of reciprocity), which may be associated with parental restriction of physical activity, were not considered. Furthermore, overall high perceived collective efficacy scores and, therefore, low variation in levels of perceived collective efficacy among parents of study participants may have reduced the ability to detect relationships between perceived collective efficacy and physical activity. Lastly, the present study did not consider participants’ length of residence in their current neighborhood. Greater length of residence may have implied increased stability within a neighborhood and greater permanence of network affiliations, which may have increased collective efficacy among neighbors.

## 5. Conclusions

Despite several limitations, this study was the first to demonstrate that parents who perceived a poor social environment (e.g., perceived collective efficacy) exhibited more constraint on their offsprings’ outdoor play in their neighborhood. Yet, constrained behaviors did not alter adolescents’ objectively measured or parent-reported physical activity. The role of the physical environment (e.g., incivilities) was not clear; yet results reveal that a better understanding of how the physical environment interacts with parental perceptions of collective efficacy and their constrained behaviors is needed. This exploratory study was the first step. Studies in larger, more diverse samples of children and adolescents are needed to understand further the role of, and the interdependence between, perceived collective efficacy and neighborhood incivilities. However, perceived collective efficacy influences parenting behaviors related to youth physical activity. Therefore, community-based programs that seek to facilitate social cohesion and control may be needed to increase adolescents’ physical activity in their neighborhoods, especially among families living in high-incivility neighborhoods. Reducing parents’ constraint of outdoor play may be achieved by targeting modifiable social environmental factors, which offers a unique opportunity to improve adolescents’ physical activity to mitigate health disparities plaguing minority and low-income populations disproportionately exposed to poor environments.

## Figures and Tables

**Table 1:** Summary scales for neighborhood incivilities and parent-reported neighborhood activity, including descriptions, scoring and internal consistency (alpha).

Variable	Total Items	Number of Items Used in the Scale	Example Items, Response Options and Scale Development (In All Cases, a Principal Component Analysis Was Used to Determine Whether Selected Items Accurately Denoted Each Variable)	Alpha	Mean (SD)	Min, Max
*Neighborhood Incivilities*						
Physical Disorder	3	No Scale	(1) garbage/litter on the street, in residential yards, commercial, businesses, institutional, industrial lots (rated 0–4: none, light, moderate, heavy)	0.3	2.6 (3.4)	0, 22
			(2) graffiti or graffiti that had been painted over on buildings or signs (rated no (0), yes (1))			
			(3) residential porches that were cluttered with personal items (rated no (0), yes (1))			
			Finding: Due to no clear factor and poor alpha levels, a physical disorder scale was not created. Items were analyzed individually.			
Physical Decay	6	3	(1) condition (rated 1–3: well-kept/good, fair, poor/badly deteriorated) of residential units	0.6	0.4 (1.4)	1, 10
			(2) condition (rated 1–3: well-kept/good, fair, poor/badly deteriorated) of residential yards			
			(3) the presence of abandoned cars (rated no (0)–yes (1))			
			Finding: A physical decay scale was created by summing all of the items resulting in a greater value indicating worse physical decay.			
Safety	4	3	(1) raters’ perceptions of whether the neighborhood was a “safe place to live” (rated 1–5; definitely safe to definitely unsafe)	0.8	4.9 (2.3)	2, 12
			(2) raters’ perceptions of whether they would feel “safe walking at night” (rated 1–5; definitely safe to definitely unsafe)			
			(3) whether the rater felt they could live in the neighborhood (rated 1–5; strongly agree to strongly disagree)			
			Finding: A safety scale was created by summing all items resulting in a greater value indicating greater danger.			
Street Safety	4	No Scale	(1) the presence of speed limit signs (rated no (0), yes (1))	−0.3	4.5 (1.0)	3, 7
			(2) presence of a bike lane (rated no (0), yes (1))			
			(3) lighting (rated 1–4: >75%, 50%–74%, 25%–49%, <25%)			
			(4) traffic volume (rated 1–4; no traffic, light, moderate, heavy)			
			Finding: Due to no clear factor and poor alpha levels, a street safety scale was not created. Items were analyzed individually.			
Overall Incivilities	5	3	garbage/litter on the street, in residential yards, commercial, businesses, institutional, industrial lots (rated 0–4: none, light, moderate, heavy), physical decay summary scale, safety summary scale, lighting on the street, traffic volume (rated 1–4; no traffic, light, moderate, heavy). Finding: An overall incivilities scale was created and included physical decay, safety and garbage/litter.	2.2	−6.4 (1.0)	−0.8, 6.1
*Parent-Reported Physical Activity*						
Neighborhood Activity	9	9	Questionnaire: “How often is your child physically active: in your driveway of alley? ... in a local street, sidewalk or vacant lot?” Options: Never (1) to 4 days/week or more (6). Recoded to indicate the number of times per month and summed. The total was dichotomized at the mean (25 times per month) to indicate physical activity in the neighborhood.	NA	17.7 (6.5)	7, 31

Not Applicable (NA) is included for the alpha value for neighborhood activity because a scale was not created and therefore, an alpha value is not applicable.

**Table 2:** Descriptive statistics of the participants by incivilities.

	Total (*n* = 71)				Low Incivilities (*n* = 41) ^a^				High Incivilities (*n* = 30) ^a^				
	Mean (SD) or *n* (%)	Median	Min	Max	Mean (SD) or *n* (%)	Median	Min	Max	Mean (SD) or *n* (%)	Median	Min	Max	*p*-Value
Age (years)	14.2 (1.6)	14.0	11.0	18.0	14.3 (1.6)	14.0	11.0	18.0	14.0 (1.5)	14.0	11.0	17.0	0.39
Male	37 (52.1)	-	-	-	22 (31.0)	-	-	-	15 (21.1)	-	-	-	0.76
Non-White	24 (33.8)	-	-	-	9 (37.5)	-	-	-	15 (62.5)	-	-	-	0.01 *
Low-Income **^b^**	20 (28.6)	-	-	-	4 (10.0)	-	-	-	16 (53.3)	-	-	-	<0.001 *
Collective Efficacy **^c^**	4.0 (0.6)	4.1	2.3	5.0	4.1 (0.4)	4.1	3.1	4.8	3.8 (0.7)	4.1	2.3	5.0	0.03 *
Cohesion	3.8 (0.6)	3.8	2.2	5.0	3.9 (0.5)	4.0	2.8	4.8	3.6 (0.7)	3.8	2.2	5.0	0.06
Control	4.1 (0.8)	4.3	1.5	5.0	4.3 (0.5)	4.2	3.3	5.0	3.9 (1.0)	4.3	1.5	5.0	0.02 *
*Constrained Behavior*													
Avoidance Behavior **^d^**	−5.7 (7.0)	−5.0	−18.0	18.0	−7.4 (6.0)	−7.0	−18.0	6.0	−3.3 (7.7)	−3.0	−18.0	18.0	0.01 *
Defensive Behavior **^e^**	−2.7 (4.7)	−4.0	−11.0	12.0	−3.8 (3.6)	−4.0	−11.0	6.0	−1.3 (5.5)	−2.0	−11.0	12.0	0.02 *
*Physical Activity*													
MVPA **^f^**	25.7 (13.7)	26.8	0.0	56.5	23.9 (13.1)	25.2	0.0	46.0	28.0 (14.5)	27.6	0.2	56.5	0.37
Neighborhood Activity **^g^**	17.7(6.5)	17.0	7.0	31.0	16.7(7.5)	16.5	7.0	30.0	19.1(7.5)	20.0	7.0	31.0	0.09
*Weight Status* **^h^**													0.09
Normal	31 (43.7)	-	-	-	22 (53.6)	-	-	-	9 (30.0)	-	-	-	-
Overweight	11 (15.5)	-	-	-	4 (9.8)	-	-	-	7 (23.3)	-	-	-	-
Obese	29 (40.8)	-	-	-	15 (36.6)	-	-	-	14 (46.7)	-	-	-	-
BMIz **^i^**	1.1 (1.0)	1.2	−1.1	2.9	0.9 (1.1)	1.0	−0.9	2.9	1.4 (0.8)	1.5	−1.1	2.6	0.03 *
Waist Circumference	84.4 (18.1)	85.2	30.0	127.0	82.4 (20.6)	79.0	30.0	127.0	87.2 (14.0)	88.1	58.0	115.0	0.27

* *p* < 0.05; independent sample *t*-test and chi-squared tests determined significant differences between low and high income participants. **^a^** Incivilities data were collected using systematic social observation at the parcel-level using Google Street View. Factor analysis was used to create a summary variable, which included physical decay, safety and litter summary variables (Table 1). The factor was then dichotomized at the median value (−0.28) to create a measure of high and low incivilities. **^b^** Low income is defined as <$40,000 annual household income. **^c^** Collective efficacy was measured using 6 questions on cohesion rated on a 5-point Likert scale (strongly disagree (1) to strongly agree (5)) and 5 questions on control rated on a 5-point Likert scale (very unlikely (1) to very likely (5)). Summary variables for cohesion and control were developed by calculating the mean across questions for each. Collective efficacy was calculated as the mean of cohesion and control summary variables. **^d^**^,**e**^ All responses were summed to compute an overall avoidance (possible range: −18–18; 9 questions) or defensive (possible range: −12–12; 6 questions) behavior score using a 5-point Likert scale (strongly disagree (−2) to strongly agree (2)) with a higher number indicating more avoidance or defensive behavior, respectively. **^f^** Moderate to vigorous physical activity (MVPA) is defined as the mean minutes measured by accelerometry. **^g^** Neighborhood activity was measured by parent-reported times per week their child was active in locations in their neighborhood (rated on a 6-point scale; never (1) to 4 days/week or more (6)). Responses were coded to indicate the number of times per month and summed. The total was dichotomized at the mean (25 times per month) to indicate physical activity in the neighborhood. **^h^** Weight status was categorized based on BMI percentile as obese (≥95), overweight (<95 and ≥85) or healthy weight (<85). **^i^** BMIz scores were calculated using the Centers for Disease Control’s statistical program for calculating BMI percentiles and z-scores using BMI, weight and height based on the child’s sex and age [32].

**Table 3:** Regression models between perceived collective efficacy (PCE) and: (1) constrained behavior (a_1_ avoidance behavior, a_2_ defensive behavior); and (2) c’ neighborhood activity, controlled for demographic variables (Figure 1).

	Diagram A								Diagram B			
	a_1_ Avoidance Behavior ^a^				a_2_ Defensive Behavior ^b^				c’ Neighborhood Activity ^c^			
	Univariate Model		Final Model		Univariate Model		Final Model		Univariate Model		Final Model	
	β (SE)	*p*	β (SE)	*p*	β (SE)	*p*	β (SE)	*p*	β (SE)	*p*	β (SE)	*p*
*Main Predictor*												
PCE **^d^**	−3.24 (1.15)	0.04 *	−2.75 (1.50)	0.07	−2.01 (1.01)	0.05 *	−1.84 (1.04)	0.08	0.14 (0.11)	0.19	0.17 (0.11)	0.14
*Demographics*												
Gender	1.73 (1.67)	0.30	1.96 (1.64)	0.24	−0.61 (1.00)	0.54	−0.57 (1.03)	0.58	0.02 (0.11)	0.84	−0.01 (0.12)	0.92
Race	2.50 (1.87)	0.19	2.26 (1.86)	0.23	−1.15 (1.25)	0.36	0.92 (1.27)	0.47	−0.12 (0.13)	0.36	−0.14 (0.14)	0.31
Income **^e^**	4.28 (2.03)	0.04 *	3.26 (2.05)	0.12	1.98 (1.42)	0.17	1.35 (1.44)	0.36	−0.00 (0.15)	0.99	0.08 (0.15)	0.62

* *p* < 0.05. **^a^**^,**b**^ All responses were summed to compute an overall avoidance (possible range: −18–18; 9 questions) or defensive (possible range: −12–12; 6 questions) behavior score using a 5-point Likert scale (strongly disagree (−2) to strongly agree (2)) with a higher number indicating more avoidance or defensive behavior, respectively. **^c^** Neighborhood activity was measured by parent-reported times per week their child was active in locations in their neighborhood (rated on a 6-point scale; never (1) to 4 days/week or more (6)). Responses were coded to indicate the number of times per month and summed. The total was dichotomized at the mean (25 times per month) to indicate physical activity in the neighborhood. **^d^** Collective efficacy was measured using 6 questions on cohesion rated on a 5-point Likert scale (strongly disagree (1) to strongly agree (5)) and 5 questions on control rated on a 5 point Likert scale (very unlikely (1) to very likely (5)). Summary variables for cohesion and control were developed by calculating the mean across questions for each. Collective efficacy was calculated as the mean of cohesion and control summary variables. **^e^** Low income is defined as <$40,000 annual household income. High income is defined as ≥$40,000 annual household income.

**Table 4:** Regression models: relationships between perceived collective efficacy and outcome variables within low and high incivilities, controlled for demographic variables.

	Low Incivilities ^a^				High Incivilities ^b^			
	Coefficients		95% CI		Coefficients		95% CI	
	β (SE)	*p*	Lower	Upper	β (SE)	*p*	Lower	Upper
*Model 1: Avoidance Behavior* **^c^**								
Collective Efficacy **^d^**	−0.13 (2.40)	0.96	−5.09	4.83	−2.94 (2.12)	0.18	−7.34	1.46
*Model 2: Defensive Behavior* **^e^**								
Collective Efficacy **^d^**	0.04 (1.66)	0.98	−3.36	3.45	−2.71 (1.69)	0.12	−6.23	0.81
*Model 6: Neighborhood Activity* **^f^**								
Collective Efficacy **^d^**	−0.06 (0.22)	0.77	−0.51	0.38	0.29 (0.12)	0.02 *	0.04	0.54

* *p* < 0.05. All models were controlled for gender, race and annual household income. **^a^**^,**b**^ Incivilities data were collected using systematic social observation at the parcel-level using Google Street View. Factor analysis was used to create a summary variable, which included physical decay, safety and litter summary variables (Table 1). The factor was then dichotomized at the median value (−0.28) to create a measure of high and low incivilities. **^c^**^,**e**^ All responses were summed to compute an overall avoidance (possible range: −18–18; 9 questions) or defensive (possible range: −12–12; 6 questions) behavior score using a 5-point Likert scale (strongly disagree (−2) to strongly agree (2)) with a higher number indicating more avoidance or defensive behavior, respectively. **^d^** Collective efficacy was measured using 6 questions on cohesion rated on a 5-point Likert scale (strongly disagree (1) to strongly agree (5)) and 5 questions on control rated on a 5-point Likert scale (very unlikely (1) to very likely (5)). Summary variables for cohesion and control were developed by calculating the mean across questions for each. Collective efficacy was calculated as the mean of cohesion and control summary variables. **^f^** Neighborhood activity was measured by parent-reported times per week their child was active in locations in their neighborhood (rated on a 6 point scale; never (1) to 4 days/week or more (6)). Responses were coded to indicate number of times per month and summed. The total was dichotomized at the mean (25 times per month) to indicate physical activity in the neighborhood.
